# Formation of zinc sulfide species during roasting of ZnO with pyrite and its contribution on flotation

**DOI:** 10.1038/s41598-018-26229-3

**Published:** 2018-05-18

**Authors:** Yong-xing Zheng, Jin-fang Lv, Hua Wang, Shu-ming Wen, Jie Pang

**Affiliations:** 1 State Key Laboratory of Complex Nonferrous Metal Resources Clean Utilization, Kunming University of Science and Technology, Kunming, 650093 China; 2 Faculty of Metallurgical and Energy Engineering, Kunming University of Science and Technology, Kunming, 650093 China; 3 Faculty of Land Resource Engineering, Kunming University of Science and Technology, Kunming, 650093 China

## Abstract

In this paper, formation of zinc sulfide species during roasting of ZnO with FeS_2_ was investigated and its contribution on flotation was illustrated. The evolution process, phase and crystal growth were investigated by thermogravimetry (TG), X-Ray diffraction (XRD) along with thermodynamic calculation and scanning electron microscopy-Energy-dispersive X-ray spectroscopy (SEM-EDS), respectively, to interpret the formation mechanism of ZnS species. It was found that ZnS was initially generated at about 450 °C and then the reaction prevailed at about 600 °C. The generated Fe_x_S would dissolve into ZnS and then form (Zn, Fe)S compound in form of Fe_2_Zn_3_S_5_ when temperature increased to about 750 °C. This obviously accelerated ZnS phase formation and growth. In addition, it was known that increasing of ZnO dosage had few effects on the decomposition behavior of FeS_2_. Then, flotation tests of different zinc oxide materials before and after treatment were performed to further confirm that the flotation performances of the treated materials could be obviously improved. Finally, a scheme diagram was proposed to regular its application to mineral processing. It was systematically illustrated that different types of ZnS species needed to be synthetized when sulfidization roasting-flotation process was carried out to treat zinc oxide materials.

## Introduction

Zinc is one of the most important metals supporting modern society. Nowadays, more than 70% of Zn is produced from zinc sulfide concentrates by conventional roasting-leaching-electrowinning processes in the world^1,2^. With continuous exploitation of resources, the primary resources are presently insufficient to supply demand. Fortunately, there are still abundant of zinc oxide resources undeveloped, such as zinc oxide ore, lead and zinc smelter slags and steelmaking dust. However, it seems to be difficult for valuable metal recovery from the refractory zinc oxide resources, which are usually characterized by low grade, complex composition and high content of slime^3,4^.

Flotation is the most common and commercial technique applied to recover nonferrous oxide minerals and for zinc recovery, sulfidization with alkali metal sulfides, followed by treatment with cationic collectors is usually adopted. After sulfidization, the hydrophilicity of the mineral surface decreases due to the presence of the sulfide ion adsorbed. In present case, the mineral can be well collected with cationic collectors^5,6^. However, its effectiveness is not entirely satisfactory when significant amounts of slime occur^3^. Moreover, it becomes almost impossible to recover zinc by conventional flotation when the zinc exists in form of amorphous glassy state, e.g. zinc in the lead smelter slag^7,8^.

In order to overcome the above disadvantages, many metallurgical methods are directly proposed. In the pyrometallurgical ones, Waelz and Ausmelt methods are commonly and industrially applied to recover zinc. But these processes need consuming considerable amounts of power and coal for providing high operating temperature (1150–1250 °C)^9^. Meanwhile, a variety of pollution caused by heavy metals, SO_2_ and waste water are produced. So, it seems not to be economically and technically feasible, especially for the low grade materials. In the hydrometallurgical ones, acid leaching and alkaline leaching are widely used to extract zinc. Acid leaching using sulfuric acid as solvent is usually considered to be effective in treating the zinc oxide materials, but there are many limitations for the materials containing silicates and basic gangues. A large quantity of silicates will dissolve and transform to gel, inhibiting the separation of the zinc sulfate solution from the slurry^10,11^. But for the basic gangues, they mainly consume considerable amounts of sulfuric acid and then transform into precipitation of calcium sulfate, making the whole process more complex and in view of this, alkaline leaching using various solvents such as ammonia, ammonium chloride and ammonium carbonate exhibits good selectivity against the basic gangues^12,13^. However, the work environment will become bad caused by the volatilization of ammonia.

It is well known that the sulfide minerals are easier to float than their corresponding oxide minerals. If an effective method to vulcanize the oxide mineral is developed, the existing mineral processing and metallurgical processes can be applied to treat these synthetic sulfides. In the available literature, mechanical-chemical^14,15^ and hydrothermal processes^16,17^ were suggested to vulcanize the zinc oxide materials, but there were some limitations in application due to slow transformation process and fine particle nature. Generally, high temperature is favorable for improving reaction rate and crystallinity^7,18^. Therefore, roasting process was proposed to vulcanize the zinc oxide materials. Li *et al*.^19^ and Zheng *et al*.^20^ investigated the sulfidization of zinc oxide mineral with elemental sulfur at high temperatures and their sulfidization extents could reach above 90%. Zheng^8^ studied mineralogical reconstruction of the lead smelter slag using pyrite as vulcanizing reagent and zinc recovery. The results showed that the sulfidization extent of zinc reached 85.62% and the zinc grade increased from 14.07% to 25.12% after one stage of flotation. However, the previous researches about sulfidization roasting have been mainly restricted to the investigation of process optimization. Studies about interaction mechanisms between zinc oxide and vulcanizing reagent and flotation responses of the treated materials are limited.

In this paper, the formation mechanisms of zinc sulfides involving evolution process, phase variation and crystal growth were investigated by TG, XRD, thermodynamic calculation and SEM-EDS. Then, flotation tests of different zinc oxide materials including natural smithsonite before and after sulfidization roasting, natural sphalerite and lead smelter slag (mainly Zn_2_SiO_4_) were carried out to further confirm that the treated zinc oxide materials exhibited good flotation responses in conventional flotation system. Finally, a systematic technical scheme was devised. The objective of this study was to clarify the formation process of zinc sulfide species at high temperatures and provide an excellent theory reference for recovering Zn from different zinc oxide resources.

## Materials and Methods

### Materials

**Figure 1:**
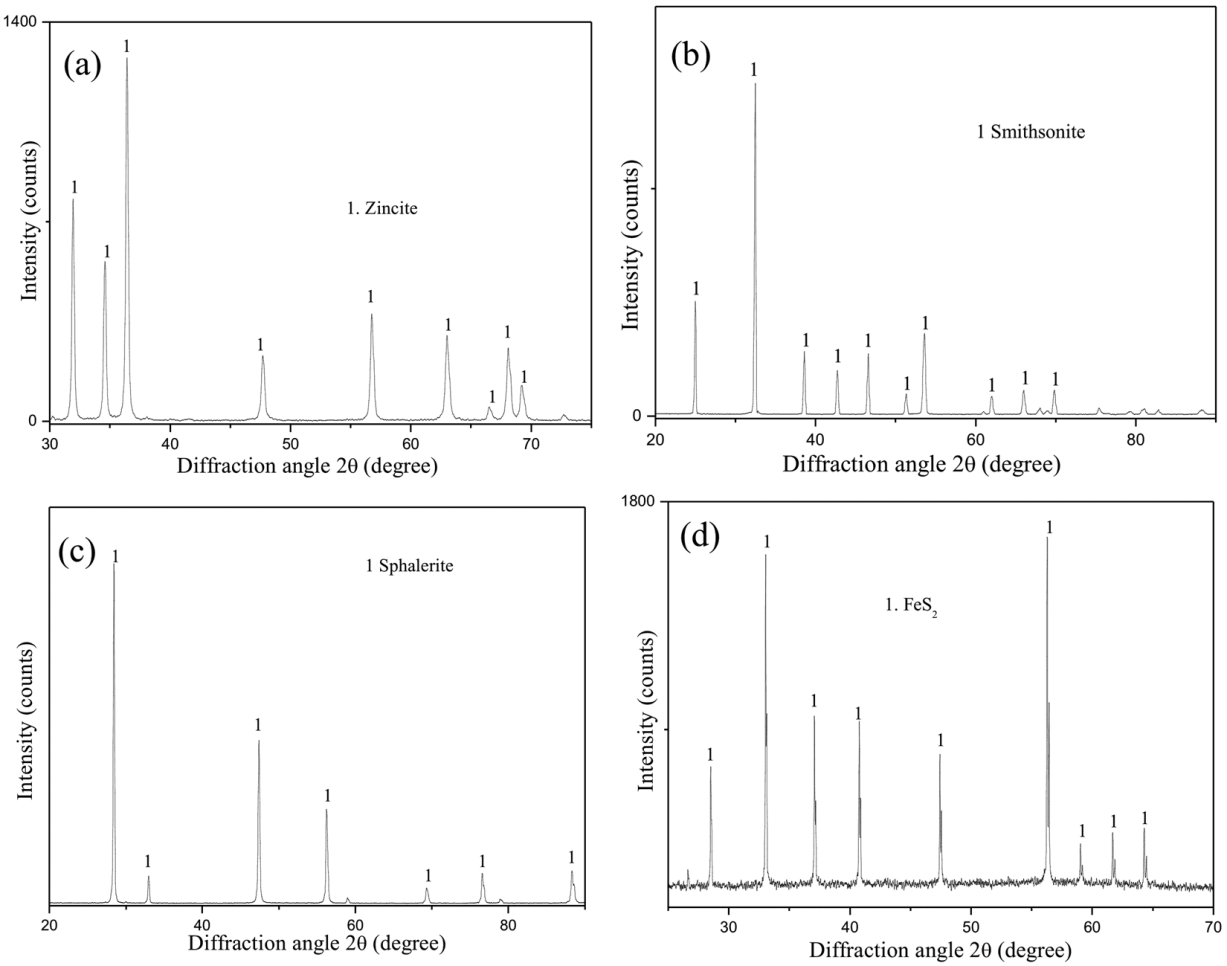
XRD patterns of the synthesized and natural samples (**a**) synthesized ZnO; (**b**) natural smithsonite; (**c**) natural sphalerite; (**d**) natural pyrite).

ZnO sample at a particle size less than 74 μm was prepared by decomposing basic zinc carbonate with an analytical grade. About 150 g of the sample was loaded into an alundum crucible equipped with a cover. Then, the pan was placed in the furnace heated at 600 °C for 1.5 h and then cooled. The obtained roasting products were analyzed by XRD, as shown in Fig. 1(a), which revealed that the sample is of high purity. Smithsonite, sphalerite and pyrite are natural crystal minerals, which were provided from a mine in Yunnan province. They were ground to −74 μm, waiting for roasting tests and various analyses. Chemical analyses^21,22^ showed that the smithsonite containing 51.24% Zn, the sphalerite containing 65.3% Zn and 32.8% S and the pyrite containing 47% Fe and 47.9% S. XRD patterns of these natural minerals are shown in Fig. 1(b–d), which also disclosed them with high purity.

Argon (Ar) was used as a protective gas with a purity of 99.9%. Copper sulphate as an activator, butyl xanthate as a collector, and terpineol as a frother were used in the micro-flotation experiments.

### Experimental method

#### TG tests

Roasting of zinc oxide with pyrite was conducted at a thermal-analyzer (NETZSCH5, STA 449 F3). Some parameters such as initial and final temperature (25~900 °C), gas flow rate, sample mass and heating rate could be exactly preset. Firstly, pyrite and zinc oxide were weighted using an electronic balance with an accuracy of 10^−4^ g, and then were mixed on an Al_2_O_3_ pan, suspended on a holder. A vertical electric furnace tube was moved downward and a vacuum of 99% was obtained. With the flow of argon from bottom of the furnace at a rate of 100 mL/min, the vacuum was slowly released to zero. The non-isothermal tests were carried out within 25 to 900 °C at a heating rate of 15 °C/min. In the isothermal tests, sample was heated at a heating rate of 30 °C/min to a desired temperature and then reacted for about 120 min. The related reactions can be described as^7,23^:

1 $$2{{\rm{FeS}}}_{2}\to 2{\rm{FeS}}+{{\rm{S}}}_{2}({\rm{g}})$$

2 $$2{\rm{ZnO}}+3{{\rm{FeS}}}_{2}=2{\rm{ZnS}}+3{\rm{FeS}}+{{\rm{SO}}}_{2}({\rm{g}})$$

From Eqs (1) and (2), it can be known that 1 mole of elemental sulfur will be lost in form of sulfur vapour (S_2_) when 1 mole of pyrite was heated. When ZnO was added into the system while the pyrite dosage was fixed as 1 mole, only 1/3 mole of elemental sulfur will be lost in form of SO_2_. In order to investigate the effect of ZnO introduced on the pyrite decomposition behaviors, which was nearly considered to be the interaction mechanism of ZnO and pyrite, the amount of pyrite (30.0 mg) was fixed while the amount of ZnO (6.75~20.25 mg) was varied in the non-isothermal process. Nevertheless, the remaining percentage outputted by the computer was based on the total amounts of zinc oxide and pyrite. Therefore, the remaining percentage relative to the amount of pyrite (30.0 mg) can be transformed as:

3 $${\rm{R}}=1-\frac{{{\rm{M}}}_{1}\times (1-{\rm{a}} \% )\times 1/2}{{{\rm{M}}}_{2}}\times 100 \% $$

where R is the remaining percentage of pyrite when the zinc oxide was added; M_1_ is the total weight of zinc oxide and pyrite, M_2_ is the weight of pyrite (30.0 mg); a% is the remaining percentage for the mixed sample of pyrite and zinc oxide; 1/2 is the weight percentage of sulfur accounting for the sulfur dioxide.

#### Pretreatment of zinc oxide minerals and flotation tests

Sulfidization roasting were usually performed in the temperature range of 600–750 °C^20,24^. In order to examine the flotation performances of zinc oxide minerals after treatment, the roasting temperature was determined as 650 °C. In our previous theoretical calculation^25^, the amount of ZnS nearly reached 100% when the FeS_2_/ZnCO_3_ mole ratio was fixed to be about 0.7 at 700 °C. Therefore, FeS_2_/ZnCO_3_ mole ratios of 0.30 and 0.15 for the surface thermal modification were selected in this paper. Pyrite and smithsonite were mixed in a desired mole ratio. The mixture was loaded into a 50 mL alundum crucible equipped with a cover. Then, the alundum crucible was placed in the furnace while the argon was introduced at a flow rate of 1.8 L/min. Finally, the heating procedure was started up until the desired temperature was obtained. After 60 min of residence time, the roasted sample was cooled under argon, waiting for flotation tests.

**Figure 2:**
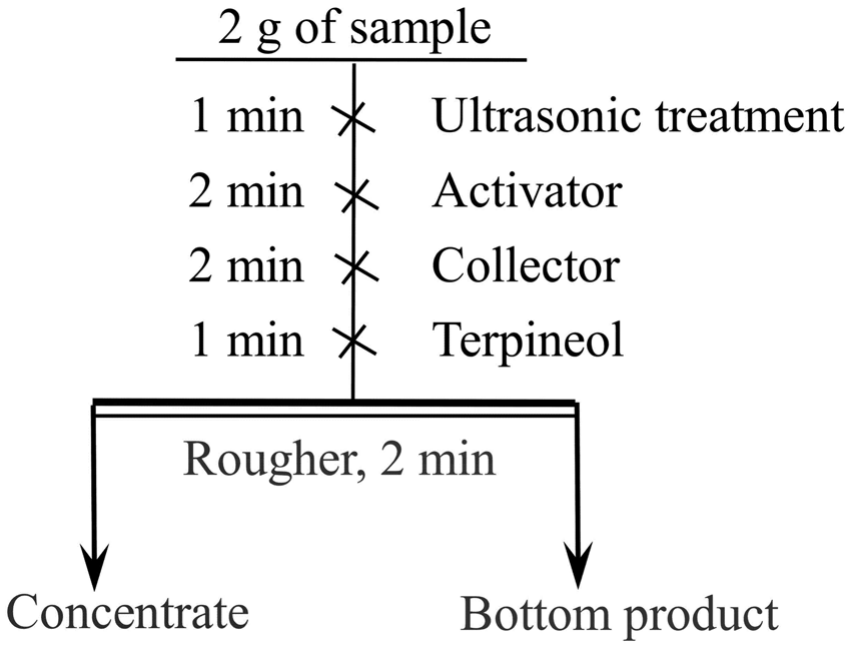
Experimental schematic of the micro-flotation.

Micro-flotation was carried out in a cell with an effective volume of approximate 40 cm^3^ and the flotation flow sheet is shown in Fig. 2. After flotation, concentrate and bottom product were washed with distilled water, filtered, dried, weighed and calculated.

### XRD and SEM-EDS analyses

The samples obtained from the isothermal tests were examined on a Bruker-axs D8 Advance XRD (Germany) with Cu Kα radiation (λ = 1.5406 Å). The operation voltage and current kept at 40 kV and 40 mA, respectively. Morphological analyses of the obtained products were detected by SEM. The SEM (JEOL.LTD, JSM-6360LV) was working at 20 kV electron accelerating voltage. Semiquantitative information analyses were also performed using an X-ray energy dispersive spectrometer (EDAX.LTD, EDX-GENESIS 60S).

## Results and Discussions

### ZnO roasting in presence of pyrite

#### TG tests

**Figure 3:**
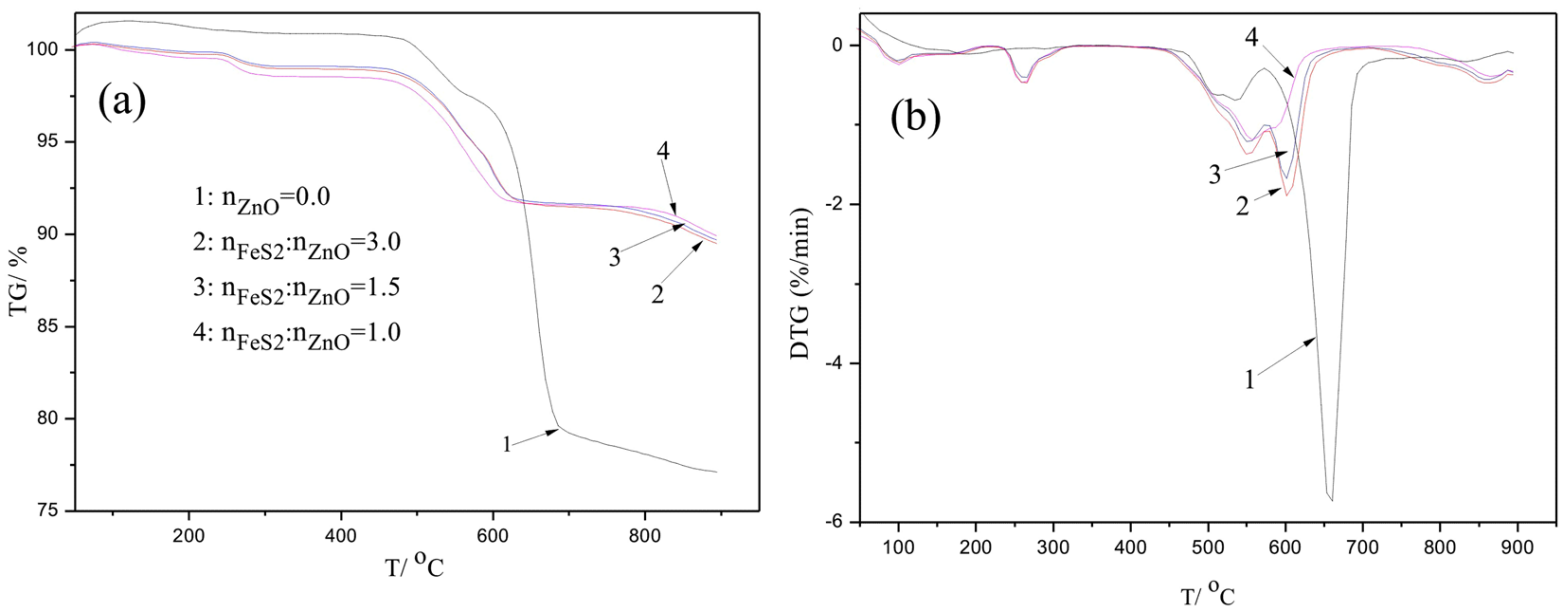
TG and DTG curves of the samples with respect to temperature and different mole ratio of FeS_2_ to ZnO.

Figure 3 shows the non-isothermal TG and DTG curves of the samples. From Fig. 3(a), it can be seen that the mass for single pyrite (Curve 1) slightly decreased when temperature increased from 480 °C to 550 °C. This may be accounted by the oxidization reaction of pyrite and adsorbed oxygen^26^. The mass slightly fluctuated as temperature increased from 550 °C to 580 °C. Mass loss continued and its maximum rate appeared at about 680 °C when temperature increased to 700 °C. These can be explained as^27,28^:

4 $$2{{\rm{FeS}}}_{2}({\rm{s}})\to 2{\rm{F}}{\rm{e}}{{\rm{S}}}_{{\rm{x}}}({\rm{s}})+(2-{\rm{x}}){{\rm{S}}}_{2}({\rm{g}})$$

5 $$2{{\rm{Fe}}}_{3}{{\rm{O}}}_{4}+5{{\rm{S}}}_{2}({\rm{g}})\to 6{\rm{F}}{\rm{e}}{\rm{S}}+4{\rm{S}}{{\rm{O}}}_{2}({\rm{g}})$$

With further increasing temperature, the mass slightly decreased, indicating that the decomposition of pyrrhotine (FeS_x_) continuously occurred (Eq. (6))^28^. These results suggested that the decomposition process of pyrite under argon atmosphere was a slow release process of sulfur from FeS_2_ to FeS.

6 $$2{{\rm{FeS}}}_{{\rm{x}}}({\rm{s}})=2{\rm{FeS}}({\rm{s}})+({\rm{x}}-1){{\rm{S}}}_{2}({\rm{g}})$$

When ZnO was introduced into the system (Curves 2–4), it can be observed that the mass slightly fluctuated and then began to decrease when temperature increased to about 450 °C. This can be explained by the solid-solid reaction (Eq. (7)).

7 $$9{\rm{F}}{\rm{e}}{{\rm{S}}}_{2}+16{\rm{Z}}{\rm{n}}{\rm{O}}\to 16{\rm{Z}}{\rm{n}}{\rm{S}}+3{\rm{F}}{{\rm{e}}}_{3}{{\rm{O}}}_{4}+2{\rm{S}}{{\rm{O}}}_{2}({\rm{g}})\,({\rm{\Delta }}{{{\rm{G}}}_{{\rm{T}}}}^{{\rm{\theta }}}=-\,133.3{\rm{k}}{\rm{J}},\,{\rm{T}}=298{\rm{K}})$$

Mass loss continued and its maximum rate (Fig. 3(b)) appeared at about 600 °C when temperature increased to about 650 °C. In addition, it was found that the remaining percentage of the sample was more than that of the single pyrite, confirming that the solid-gas reaction occurred (Eq. (8))^20,24^. In other words, the released elemental sulfur from pyrite was fixed in form of ZnS, resulting in mass increase of the remaining sample.

8 $$4{\rm{Z}}{\rm{n}}{\rm{O}}({\rm{s}})+3{{\rm{S}}}_{2}({\rm{g}})=4{\rm{Z}}{\rm{n}}{\rm{S}}({\rm{s}})+2{\rm{S}}{{\rm{O}}}_{2}({\rm{g}})$$

Additionally, mass loss of the sample still continued when the temperature increased above 650 °C. This may be attributed to the decomposition of pyrrhotine (FeS_x_) (Eq. (6)) and its further reaction with ZnO. However, it seemed that increasing of ZnO dosage had few effects on the decomposition behavior of pyrite, indicating that its capacity of fixing sulfur was limited.

**Figure 4:**
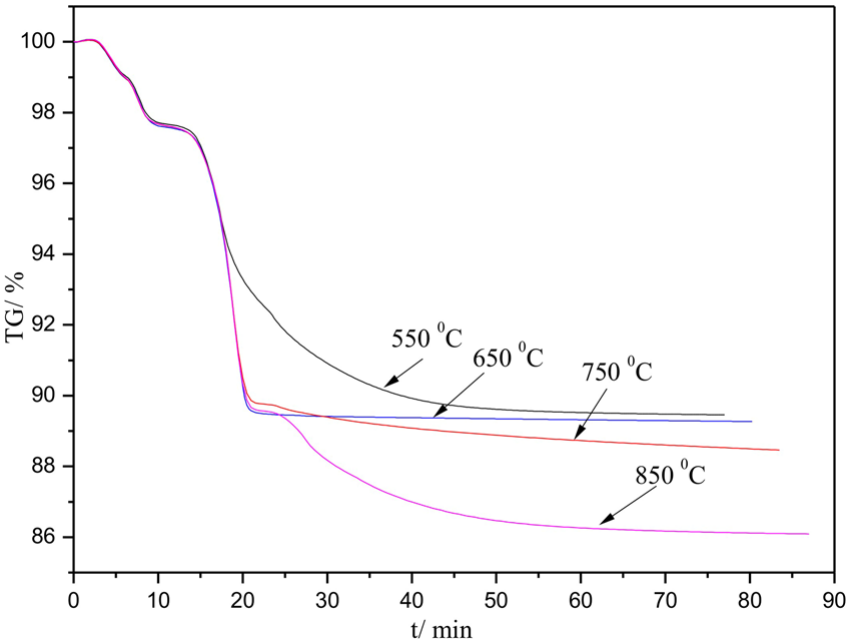
TG curves of the sample with respect to time and temperature (n_FeS2_:n_ZnO_ = 1:1).

Figure 4 shows the isothermal TG curves of the sample. It can be seen that mass loss slowly decreased in the time range of 15–50 min when temperature was fixed as 550 °C. When the temperature increased, the mass loss sharply decreased in the time range of 15–20 min. Combining with the TG curves (Fig. 3), it can be known that the mass loss was mainly attributed to the interaction of zinc oxide and the generated sulfur vapor (Eq. (8)). With further prolonging their holding time, the mass was nearly constant at 550 °C and 650 °C, but the the mass decreased at 750 °C and 850 °C, indicating that the further decomposition of pyrrhotine (FeS_x_) occurred (Eq. (6)). In addition, it can be also observed that the temperature was the higher, the less the remaining sample mass was. This can be explained that the decomposition of pyrite was accelerated when the temperature increased.

#### Phase transformation

**Figure 5:**
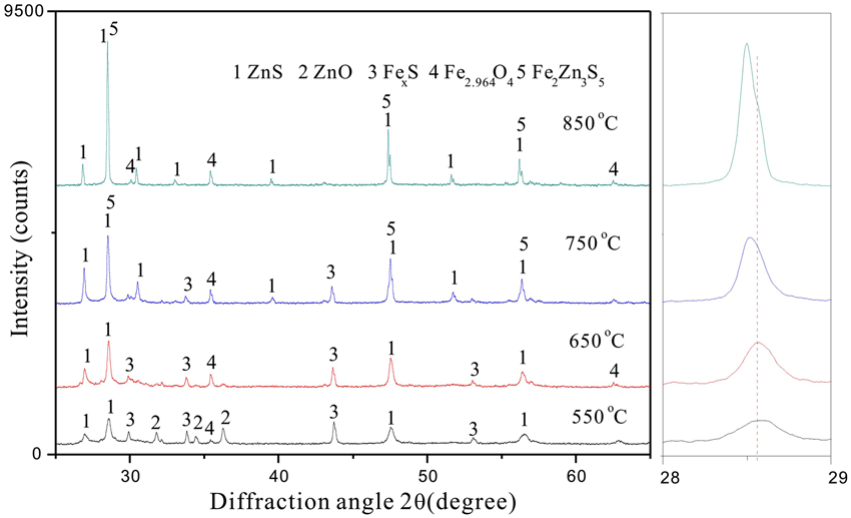
XRD patterns of the roasted products at different temperatures (n_FeS2_:n_ZnO_ = 1:1).

In order to confirm the above deductions, phase analyses were carried out for the obtained products under isothermal TG tests and the results are shown in Fig. 5. According to this figure, it can be seen that there were obvious peaks of ZnS and Fe_x_S, weak peaks of ZnO and Fe_2.964_O_4_ at 550 °C, indicating that the reactions involving Eqs (4), (7) and (8) occurred. The peak of ZnO disappeared and the peak intensity of ZnS increased when the temperature increased to 650 °C. With further increasing temperature, the peak intensity of ZnS further increased and interestingly, the Fe_x_S peak completely disappeared at 850 °C. Moreover, the FeS peak which was generated by the further decomposition of Fe_x_S (Eq. (6)) couldn’t be also observed. This may be explained that the ZnS and FeS formed the (Zn, Fe)S compound.

Additionally, it can be also observed that the (111) peak shifted towards low diffraction angle areas with increasing roasting temperature, indicating that the Fe^2+^ in FeS replaced the Zn^2+^ in ZnS and then formed the (Zn, Fe)S compound in form of Fe_2_Zn_3_S_5_. As Fe^2+^ in the ilmenite have larger ionic radius than that of Fe^3+^ generated by magnetic modification, the unite cell exhibits continuous expansion with increasing roasting temperature^29–31^, corresponding to the change in (111) peak position.

**Figure 6:**
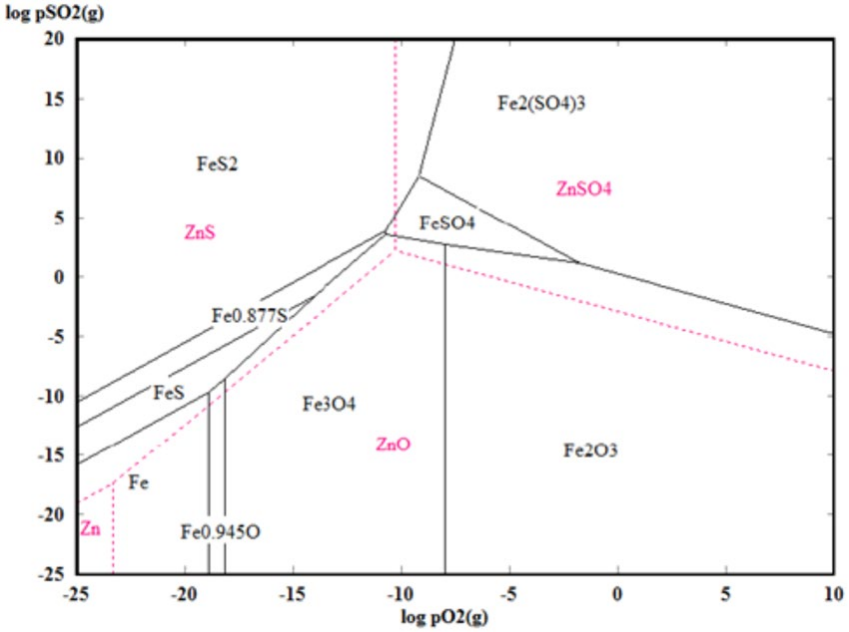
Predominance area diagrams of Zn-S-O and Fe-S-O system at 800 °C.

In order to gain insight into the phase transformation process, the phase stability boundaries were calculated using the Tpp Diagrams module of Out-okumpu HSC5.0^25,32^, assuming that all solids have a unit activity. The predominance area diagram of Zn-S-O (red dot line) and Fe-S-O (black solid line) system at 800 °C were plotted, as shown in Fig. 6. It can be seen that the condensed phases were obviously affected by the partial pressures of O_2_ and SO_2._ In this study, the desired ZnS could be prepared using ZnO and FeS_2_ as raw material by controlling their partial pressures. In addition, it can be observed that other phases such as Fe, FeO, Fe_3_O_4_, FeS, Fe_0.877_S and FeS_2_ could exist with ZnS. Pyrite (FeS_2_) would be decomposed into Fe_x_S in practice, but it would be difficult to further transform into metallic Fe. On the other hand, FeS_2_ and Fe_x_S would be also oxidized into Fe_3_O_4_. Therefore, it was reasonable that only the peaks of Fe_3_O_4_ and Fe_x_S were detected by XRD.

#### Morphology changes

**Figure 7:**
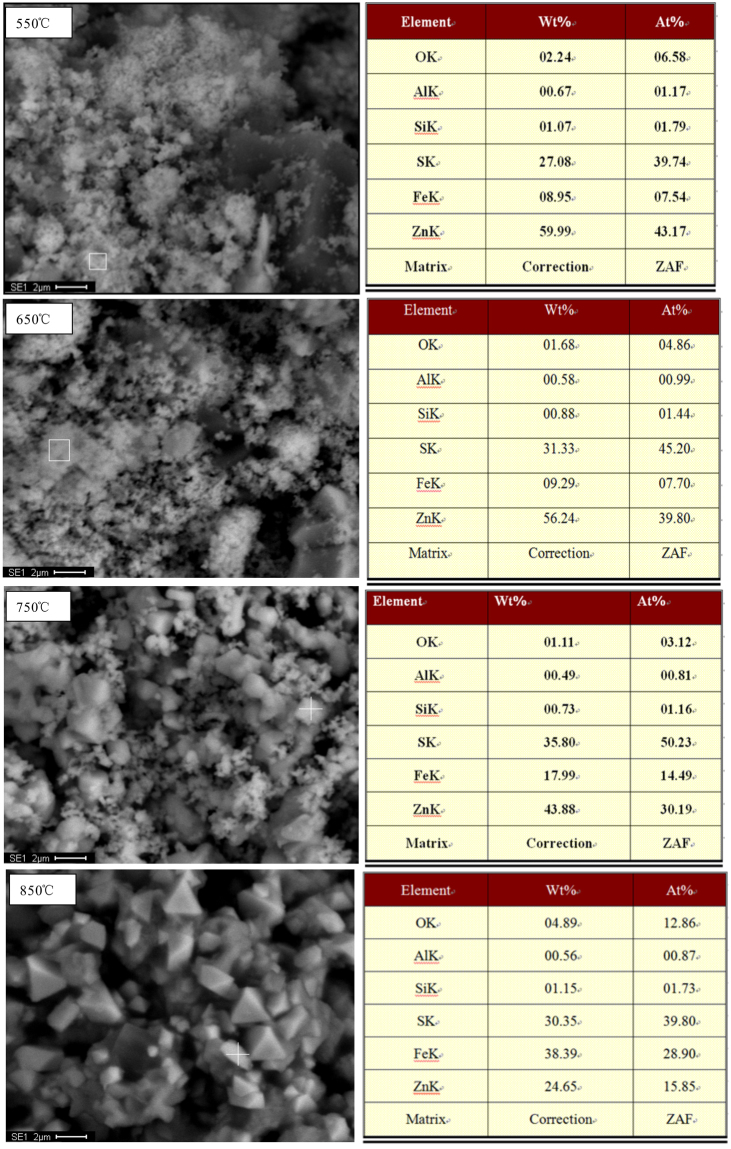
BSE images and EDS spectrums of the roasted products at different temperatures.

It was surprising that the phase of Fe_x_S disappeared when roasting temperature increased to 850 °C. In order to further clarify this phenomenon, the obtained samples under isothermal TG tests were examined by SEM-EDS and their corresponding results are shown in Fig. 7. It can be seen that the ZnS particles with cotton wool configuration were formed at 550 °C. There was no obvious change when the temperature increased to 650 °C. With further temperature increasing, the ZnS particles started to aggregate and grow, resulting in occurrence of some grains with tetrahedron and kidney shapes, which exhibited complete crystal nature. This may be accounted by the explanation that the molten phase is responsible for the rapid grain coarsening, where smaller particles will go into solution preferentially and precipitate on larger particles, accelerating the transport rate as liquids diffuse faster than solids^8,33,34^. In addition, the EDS spectrums of the roasted samples also exhibits that the Fe content increased with increasing the roasting temperature, confirming that the ZnS and FeS formed the (Zn, Fe)S compound. This result explained that the Fe_x_S phase could not be detected by XRD. Besides, the elemental sulfur content also increased with increasing the temperature, corresponding to the peak intensity of ZnS increased in Fig. 5.

Therefore, pyrite can be considered as an effective vulcanizing agent for sulfidization of ZnO. On the one hand, it can not only provide sulfur at high temperatures, but also serve as an activating agent (mainly FeS) to accelerate the formation of ZnS phase. However, it is not favorable for the following flotation when excessive dosage of FeS_2_ is added due to the increase of Fe content in (Zn, Fe)S compound. In addition, the un-reacted Fe_x_S can easily report the flotation concentrate in the process of zinc recovery, making the whole separation process more complex.

### The effect of above pretreatment on flotation

#### Surface sulfidization and flotation responses

**Figure 8:**
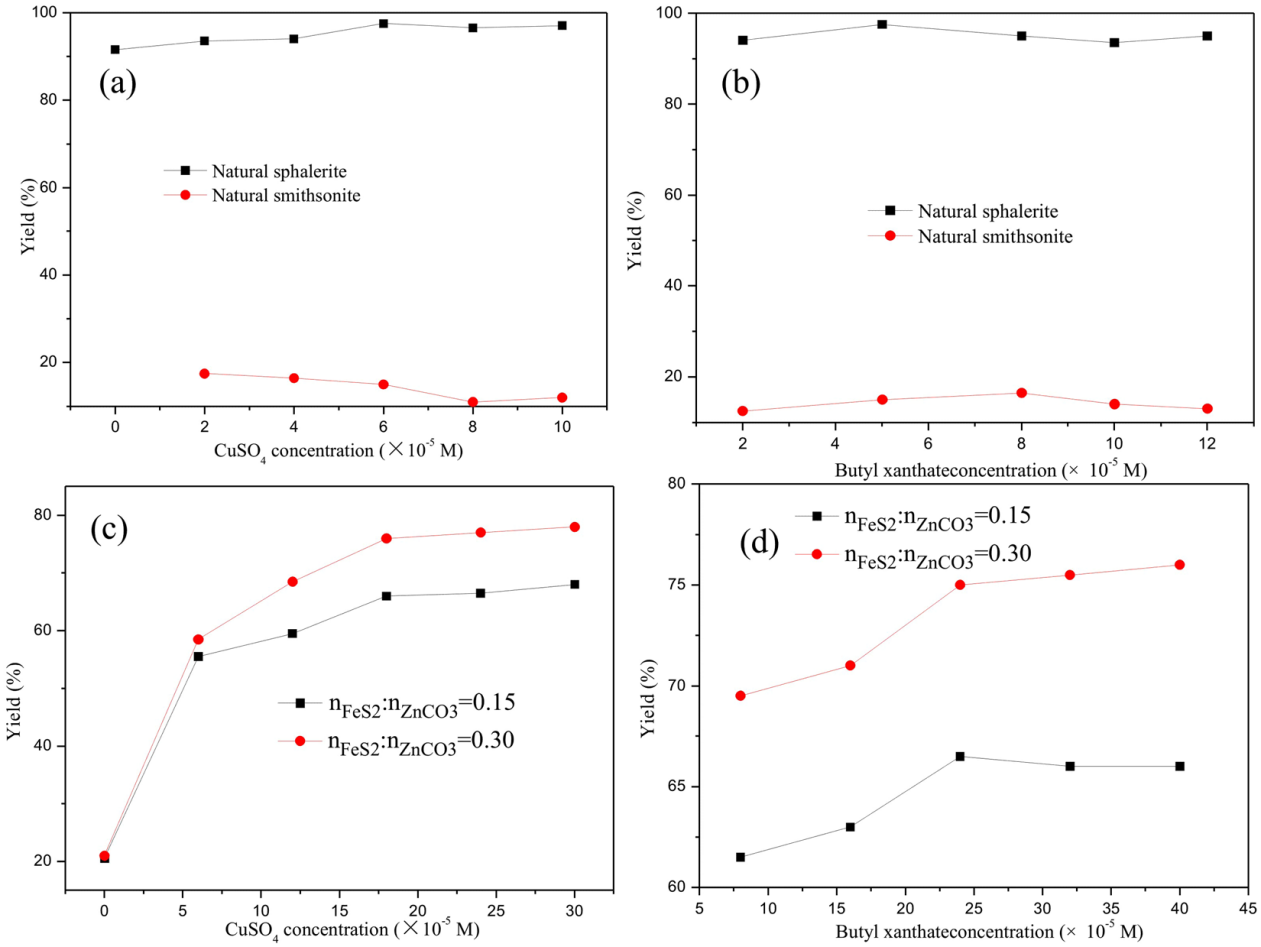
Flotation performances of natural smithsonite before and after treatment and natural sphalerite at a natural pH and a terpineol dosage of 5 × 10^−5^ M (**a**) butyl xanthate concentrations: 5 × 10^−5^ M; (**b**) copper sulphate concentrations: 6 × 10^−5^ M; (**c**) butyl xanthate concentrations: 40 × 10^−5^ M; (**d**) copper sulphate concentrations: 18 × 10^−5^ M).

It is technically feasible to recover valuable metals from the refractory oxides resources by sulfidization roasting followed by flotation. In the available literature, studies mainly focused on improving sulfidization extent of ZnO materials^19,24,34^. Factually, it is not fully advisable that the flotation response is an absolute positive correlation with its sulfidization extent. It is well known that copper and lead oxide minerals, except zinc oxide minerals, after treatment with Na_2_S can be well collected by xanthate^5,35^. The poor zinc recovery is mainly ascribed to a terrible surface sulfidization of ZnO mineral in the pulp. Combining with the above analyses, a stable thin film of ZnS could be formed on the surface of the ZnO mineral by sulfidization roasting with pyrite in a temperature range of 450–750 °C. In order to confirm that the zinc oxide mineral after sulfidization roasting can be recovered by conventional flotation technology, micro-flotation tests were carried for different minerals, as shown in Fig. 8.

From Fig. 8(a), it can be seen that flotation yield of natural sphalerite increased to 97.5%, whereas flotation yield of smithsonite only reached 15% when copper sulphate concentration increased to 6 × 10^−5^ M. Increasing of butyl xanthate concentrations also had little effects on the flotation yield of smithsonite (Fig. 8(b)). After sulfidization roasting with pyrite at different dosages, their flotation performances had obviously changed. From Fig. 8(c), it can be observed that flotation yield of the treated smithsonite increased from 20.5% to 66% and from 21% to 76%, respectively corresponding to the FeS_2_/ZnCO_3_ mole ratios of 0.15 and 0.30, when copper sulphate concentration increased from 0 M to 18 × 10^−5^ M. With further increasing copper sulphate concentration, their flotation yields had little changes. Therefore, the optimal copper sulphate concentration was determined to be 18 × 10^−5^ M and the following experiments were carried out at this concentration. The effect of butyl xanthate concentrations on their flotation yields were shown in Fig. 8(d). It can be seen that their flotation yields reached 66.5% and 75% when butyl xanthate concentrations increased 24 × 10^−5^ M. With further increasing butyl xanthate concentration, there were little changes for their flotation yields. Compared with the natural sphalerite, the flotation yield seemed to be lower. This may be accounted by the fact that not only ZnS films formed on the surface of zinc oxide minerals, but also iron oxides such as Fe_3_O_4_ appeared after roasting. The latter could not be collected by the xanthate, resulting in the lower flotation yield of the mixed material. In other word, the flotation yield for the separate smithsonite after treatment should be higher than 75%. Therefore, it was concluded that the smithsonite after sulfidization roasting had a similar flotation behavior to the natural sphalerite. In addition, smithsonite roasted with pyrite at a FeS_2_/ZnCO_3_ mole ratio of 0.3 exhibited a good flotation performance contrast to the sulfidization treatment at a FeS_2_/ZnCO_3_ mole ratio of 0.15, further verifying the moderate FeS_2_/ZnCO_3_ mole ratio (0.3) in our previous studies about surface modification of smithsonite at high temperatures^25^.

#### Complete sulfidization and flotation responses

It is very necessary to completely transform the ZnO mineral at a fine size fraction into ZnS mineral and even though, the artificial ZnS mineral can be also difficult to be collected by xanthate due to the fine particle nature^7,8,34^. Taking the lead smelter slag for example, zinc oxide mainly existed in form of Zn_2_SiO_4_^7,8^. It is usually difficult to transform the Zn_2_SiO_4_ into ZnS in the temperature range of 450–750 °C. This can be explained that the sulfidization of Zn_2_SiO_4_ needed more rigorous thermodynamic conditions than that of the common ZnO.

In our previous studies^7,8^, it was found that temperature had an obvious affect on the sulfidization extent of Zn_2_SiO_4_. The results showed that the sulfidization extent of Zn_2_SiO_4_ increased from about 21% to 83%, when the temperature increased from 550 °C to 850 °C. In addition, it was also confirmed that the (Zn, Fe)S compound could be more easily formed at a higher temperature, which obviously facilitated the aggregation of ZnS particles. After one stage of flotation for the materials roasted at 850 °C, the zinc grade and recovery increased from about 14% to 25% and from 0 to 67%, respectively.

#### Contribution on flotation

**Figure 9:**
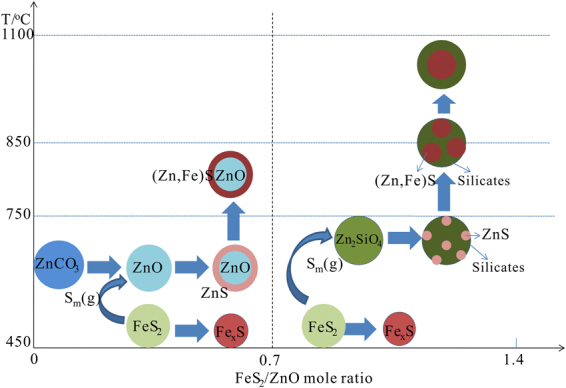
Proposed scheme diagram for sulfidization of refractory zinc oxides materials.

In summary, different types of ZnS species need to be synthetized when sulfidization roasting-flotation process is carried out to treat different zinc oxide materials. This significantly depends on the roasting temperature and pyrite dosage. In order to regularize the application of sulfidization roasting to mineral processing, a scheme diagram for the sulfidization of refractory zinc oxides materials was proposed, as shown in Fig. 9.

According to the figure, it can be known that recovery of zinc from different zinc materials needed different sulfidization methods. When zinc recovery from the zinc oxide ores, where the zinc mainly exists in form of carbonate, is carried out, surface sulfidization will be reasonable. On the one hand, the FeS_2_/ZnCO_3_ mole ratio should be lower than 0.7, which is the critical value in theory for complete transformation of ZnO into ZnS^25^. On the other hand, the roasting temperature should be controlled in the range of 450–750 °C. when the FeS_2_/ZnCO_3_ mole ratio nears to 0.7 and even beyond this value, the remaining iron sulfides also reports the flotation concentrate. In addition, when the roasting temperature beyond 750 °C, the generated ZnS mineral not only aggregated with Fe_x_S, followed by formation of (Zn, Fe)S compound, but also aggregated with other gangues. Both of which were not beneficial for improving grade of the flotation concentrate. When zinc recovery from the zinc silicate materials is performed, it is necessary to completely transform zinc silicates to zinc sulfides. On the one hand, the FeS_2_/ZnO mole ratio should be higher than 0.7. On the other hand, the roasting temperature should be higher than 750 °C. According to Fig. 7 and our previous studies^7,8^, the roasting temperature should be around 850 °C. The (Zn, Fe)S compound could be more easily formed at this temperature, accelerating the aggregation of ZnS particles.

## Conclusions

1. Formation of zinc sulfide species during roasting of ZnO with pyrite was accompanied by the decomposition and transformation of FeS_2_. Many species such as ZnS, (Zn, Fe)S compound in form of Fe_2_Zn_3_S_5_, Fe_x_S and Fe_2.964_O_4_ were formed in the process. Pyrite could not only provide sulfur at high temperatures, but also serve as an activating agent (mainly FeS) to accelerate the formation of ZnS phase.
2. Zinc sulfide was initially generated at about 450 ^o^C and then the sulfidization reaction prevailed at about 600 ^o^C. The generated Fe_x_S would dissolve into ZnS and then form (Zn, Fe)S compound in form of Fe_2_Zn_3_S_5_ when temperature increased to about 750 ^o^C. With further increasing temperature, the ZnS particles obviously aggregated, making the particle size increase. In addition, increasing of ZnO dosage had few effects on the decomposition behavior of pyrite.
3. Zinc recovery from different zinc oxide materials by sulfidization roasting-flotation process was carried out. Flotation yield of the natural smithsonite after roasting with pyrite at a FeS_2_/ZnCO_3_ mole ratio of 0.30 increased by about 55%, contrast to the un-treated natural smithsonite. The zinc silicates in the lead smelter slag could be well transformed into zinc sulfides and the zinc flotation performances were obviously improved.
4. A scheme diagram for the sulfidization of refractory zinc oxides materials was proposed to regularize the application of sulfidization roasting to mineral processing. Different types of ZnS species needed to be synthetized when sulfidization roasting-flotation process was carried out to treat different zinc oxide materials. The formation of ZnS species mainly depended on the roasting temperature and pyrite dosage.
